# Network analysis identifies circulating miR-155 as predictive biomarker of type 2 diabetes mellitus development in obese patients: a pilot study

**DOI:** 10.1038/s41598-023-46516-y

**Published:** 2023-11-09

**Authors:** Giuseppina Catanzaro, Federica Conte, Sofia Trocchianesi, Elena Splendiani, Viviana Maria Bimonte, Edoardo Mocini, Tiziana Filardi, Agnese Po, Zein Mersini Besharat, Maria Cristina Gentile, Paola Paci, Susanna Morano, Silvia Migliaccio, Elisabetta Ferretti

**Affiliations:** 1 Department of Experimental Medicine, Sapienza University of Rome, Policlinico Umberto I, Viale Regina Elena 324, 00161 Rome, Italy; 2 Institute for Systems Analysis and Computer Science “A. Ruberti” (IASI), National Research Council (CNR), 00185 Rome, Italy; 3 Department of Movement, Human and Health Sciences, University of Foro Italico, 00135 Rome, Italy; 4 Department of Molecular Medicine, Sapienza University, 00161 Rome, Italy; 5 Department of Computer, Control and Management Engineering, Sapienza University, 00161 Rome, Italy

**Keywords:** Biomarkers, Diseases, Endocrinology

## Abstract

Obesity is the main risk factor for many non-communicable diseases. In clinical practice, unspecific markers are used for the determination of metabolic alterations and inflammation, without allowing the characterization of subjects at higher risk of complications. Circulating microRNAs represent an attractive approach for early screening to identify subjects affected by obesity more at risk of developing connected pathologies. The aim of this study was the identification of circulating free and extracellular vesicles (EVs)-embedded microRNAs able to identify obese patients at higher risk of type 2 diabetes (DM2). The expression data of circulating microRNAs derived from obese patients (OB), with DM2 (OBDM) and healthy donors were combined with clinical data, through network-based methodology implemented by weighted gene co-expression network analysis. The six circulating microRNAs overexpressed in OBDM patients were evaluated in a second group of patients, confirming the overexpression of miR-155-5p in OBDM patients. Interestingly, the combination of miR-155-5p with serum levels of IL-8, Leptin and RAGE was useful to identify OB patients most at risk of developing DM2. These results suggest that miR-155-5p is a potential circulating biomarker for DM2 and that the combination of this microRNA with other inflammatory markers in OB patients can predict the risk of developing DM2.

## Introduction

Obesity is a common condition which is consistently linked to an increased risk of developing a wide range of metabolic chronic disorders, such as metabolic syndrome (MS), type 2 diabetes (DM2), cardiovascular diseases (CVD), non-alcoholic fatty liver disease (NAFLD), musculoskeletal diseases and some cancers^1–5^.

In particular, it is well known that DM2 is one of the major concerns in individuals affected by obesity, due to the strong link between the two disorders, which has even brought to define with the new term “diabesity” the unhealthy association of obesity and DM2^6^. Indeed, both obesity and DM2 global cost burden and its social consequences have dramatically increased in the last decades, and they will further increase by 2030^6^.

The markers used in clinical practice for the determination of metabolic alterations do not allow the identification of the subjects most at risk of developing complications, such as DM2. MicroRNAs are short, non-coding RNA molecules, that negatively modulate gene expression at post-transcriptional level. Through their ability to influence protein translation, microRNAs have emerged as powerful regulators of many different biological processes^7^. Moreover, due to their stability and readily detectability in blood, circulating microRNAs have emerged as potential biomarkers for many pathological processes, including metabolic chronic diseases^8^. The recent use of microRNAs as circulating biomarkers represents an innovative and sensitive approach for the early screening of individuals at risk of several disorders, including non-communicable diseases^8–11^.

Previous studies investigated the connection between microRNAs and risk factors connected to MS, such as DM2, hypertension, dyslipidemia and obesity. In a recent systematic review, Brandao Lima et al. summarized the links between circulating microRNAs and the main risk factors for MS^12^. Of note, miR-122, miR-221, miR-222 and miR-423 were related with adiposity, lipid and glycemic metabolism^12^. In older overweight or obese adults with DM2 miR-21, miR-27a, miR-30d and miR-155 presented a negative relationship with total and central body obesity^13–15^, whilst miR-101 presented a positive relationship. Another recent systematic review conducted by Solis-Toro et al. identified 12 microRNAs, namely miR-505-5p, miR-148a-3p, miR-19b-3p, miR-320b, miR-342-3p, miR-197-3p, miR-192-5p, miR-122-5p, miR-103, miR-130a, miR-155-5p and miR-375, as potential biomarkers for metabolic risk^16^. Additionally, another meta-analysis reported 7 microRNAs (miR-142-3p, miR-140-5p, miR-222, miR-21-5p, miR-221-3p, miR-125-5p, miR-103-5p) as dysregulated in OB subjects, while two of them (miR-142-3p and miR-222) were concordantly up-regulated also in DM2 patients^17^. Since dyslipidemia as well as hypertension and DM2 are among the main risk factors for the development of cardiovascular disease (CVD), a number of studies focused on the circulating microRNAs able to predict cardiovascular events^18^. MiR-126, miR-197 and miR-223 were significantly associated with the risk of myocardial infarction^19^ as well as miR-92a, whose upregulation in DM2 patients appear years before the development of coronary artery disease (CAD)^20^. Additionally the combination of five microRNAs (miR-106a-5p, miR-424-5p, let-7g-5p, miR-144-3p and miR-660-5p) was proposed to be used for myocardial infarction prediction in healthy individuals^21^.

Thus, aim of the present research was to evaluate and to determine a specific microRNA pattern, present in obese individuals affected by DM2, which could identify obese subjects at increased risk of developing this chronic metabolic disease.

## Results

### MicroRNA profiling of the discovery cohort patients

We evaluated the expression profiles of 798 microRNAs derived from EVs and total plasma (TP) of a group of 12 obese patients (OB), 10 obese patients with DM2 diagnosis (OBDM) and 9 normal body weight donors (HD). Data from EVs were available for 8 out 9 HD. Patients’ clinical features are reported in Table 1.

**Table 1:** Clinical features of the discovery cohort patients'.

	Discovery cohort			*p*-value
	HD (n = 9)	OB (n = 12)	OBDM (n = 10)	
Age	56.11 ± 4.65	56.33 ± 4.56	62.50 ± 9.05	0.0512
Gender males/females (n)	5/4	9/3	5/5	NA
BMI	24.17 ± 2.62	42.00 ± 8.62	36.27 ± 4.51	< 0.0001°°°°; 0.0018^§§^
Smoking habit Yes/No/Ex (n)	NA	1/8/2	2/5/2	NA
Waist circumference	NA	120.25 ± 11.67	120.56 ± 12.43	0.95
Systolic BP	123.33 ± 8.66	127.50 ± 11.58	137.50 ± 18.89	0.0820
Diastolic BP	80.00 ± 5.00	72.08 ± 3.96	77.50 ± 11.61	0.0611
Glycemia	92.66 ± 6.44	94.92 ± 8.89	145.74 ± 29.17	< 0.0001^§§§§^; < 0.0001****
HbA1c	NA	5.70 ± 0.36	7.78 ± 1.44	0.0007***
HOMA-I	NA	4.54 ± 2.46	7.83 ± 4.22	0.0467*
Insulin resistance Yes/No (n)	NA	8/3	8	NA
HDL cholesterol	71.22 ± 15.39	43.82 ± 5.91	48.57 ± 10.03	< 0.0001°°°°; 0.0005^§§§^
Triglycerides	102.67 ± 50.45	140.64 ± 63.00	162.11 ± 79.51	0.1593

HD, Healthy donors; OB, obese patients; OBDM, obese patients affected by type 2 diabetes (DM2); BMI, body mass index; BP, blood pressure. *p < 0.05, ***p < 0.001 OB versus OBDM, ****p < 0.0001 OB versus OBDM, °°°°*p* < 0.0001 HD versus OB, ^§§^*p* < 0.01 HD versus OBDM, ^§§§^*p* < 0.001 HD versus OBDM, ^§§§§^*p* < 0.0001 HD versus OBDM.

We combined the microRNA expression data with the clinical data of patients by exploiting the network-based methodology implemented by the WGCNA software^22,23^. This approach first builds a correlation network and searches for network modules of microRNA expression. Then, the weighted average of the microRNA expression profiles of each module is summarized by using the Module Eigengene (ME) and, to relate each ME with the clinical outcome, the clinical data for each patient are used as external sample traits to be incorporated into the co-expression network. Finally, the module-trait association is evaluated by computing for each module the correlation and the statistical significance (*p*-value) between its ME and each external sample trait.

#### WGCNA on EVs data

**Figure 1:**
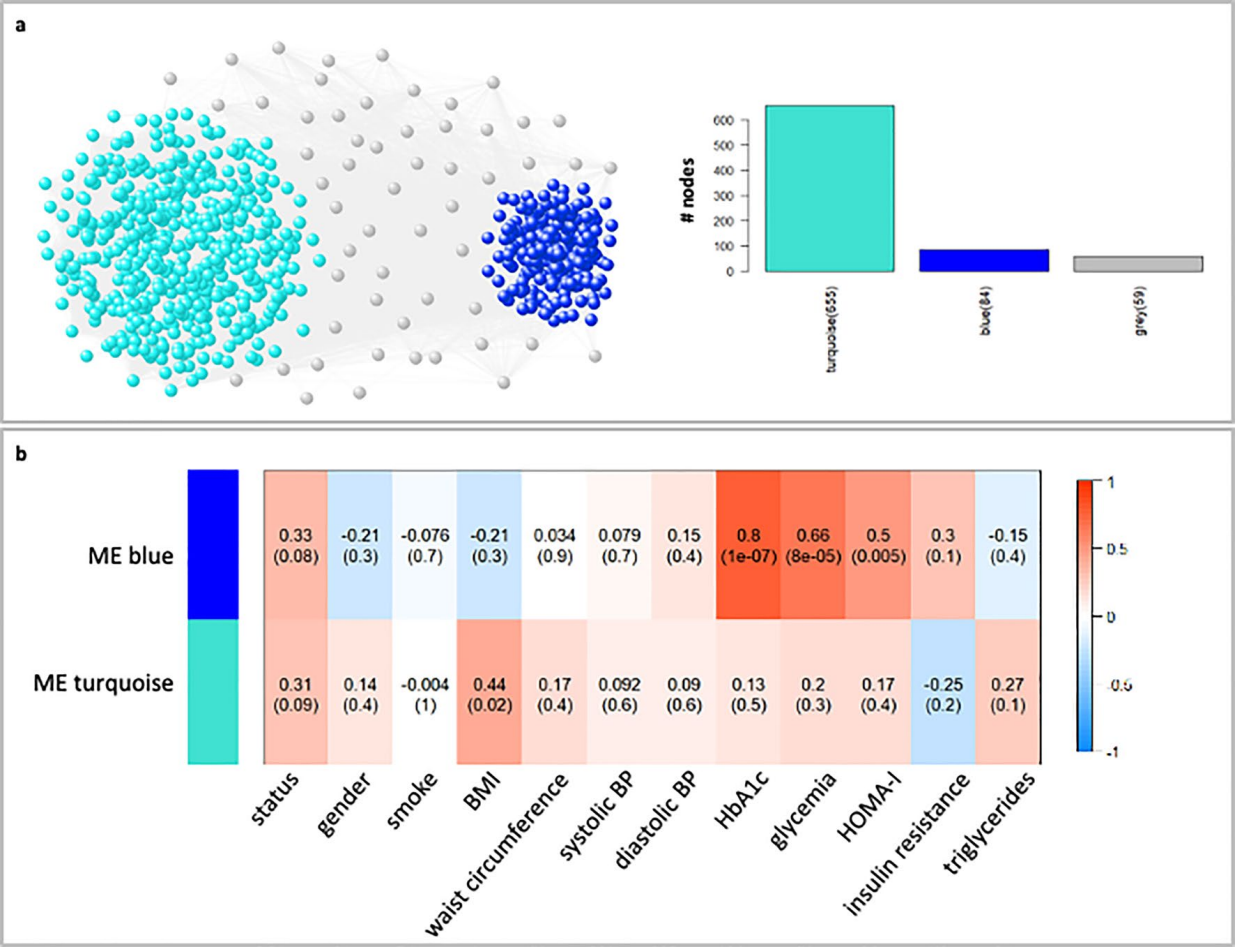
WGCNA analysis on EV data. (**a**) *WGCNA network*. In the correlation network (left), the WGCNA detected modules are highlighted and colored according to the corresponding module labels. Grey color was used to indicate nodes that could not be classified into any modules. In the bar plot (right), bars represent the size of each WGCNA detected module and are colored according to the corresponding module labels. (**b**) *Module-trait associations*. In the heatmap, each row corresponds to a module eigengene and each column to a clinical trait of interest. Each cell contains the corresponding correlation and *p*-value. The heatmap is color-coded by correlation according to the color legend.

The WGCNA analysis performed on circulating microRNAs derived from EVs led to a co-expression network made of two well-defined modules with the size of 84 and 655 microRNAs (Fig. 1a, Supplementary Table 2).

**Figure 2:**
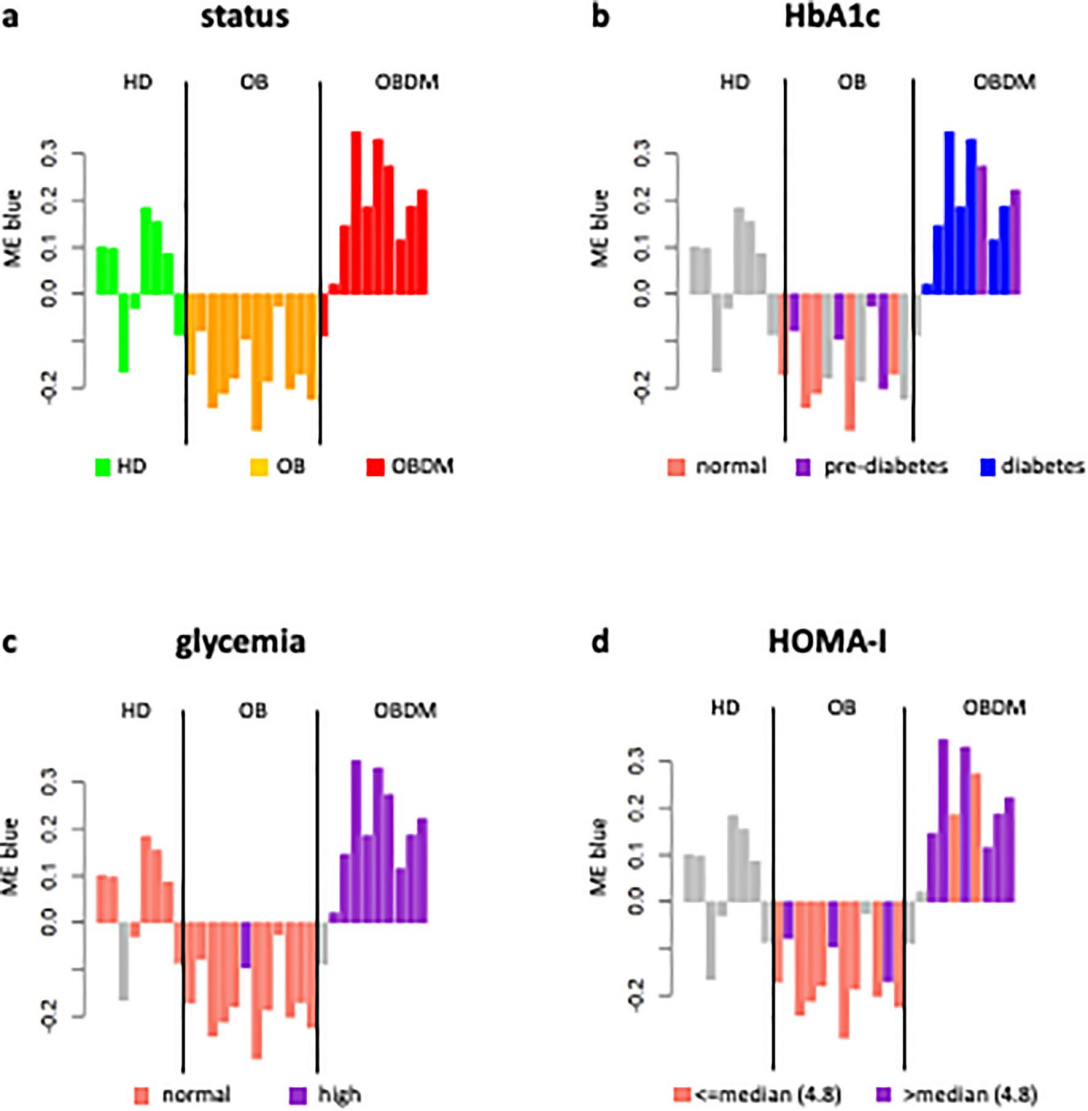
Module-trait association for EV data. Bar plots of the expression levels (y-axis) of blue module eigengene across healthy, OB, and OBDM samples (x-axis). Expression levels of the module eigengenes were log2-transformed and z-score normalized. In each panel, bars were colored according to the stratification used for the specific clinical trait of interest. For HOMA-I, the samples were stratified according to the corresponding median value only for display purposes. Grey was used to indicate not available data.

The heatmap of the module-trait association (Fig. 1b) shows how the blue module, although with a *p*-value slightly higher than the standard significance level (i.e., 0.05), exhibits the strongest association with the individuals’ status, treated as categorical variable, that increases moving from healthy to diseased condition. Specifically, we considered three levels for the variable “status” (1 = HD, 2 = OB, 3 = OBDM). The positive sign of this correlation indicates that high levels of microRNAs are a sign of pathological conditions and, in particular, of the OBDM status (Fig. 1b and Fig. 2a). In addition, the ME blue was found to exhibit a high (statistically significant) positive correlation with traits called HbA1c, glycemia and HOMA-I (Fig. 1b). It is worth noting that, in the current study, we investigated the relationship of the available clinical variables with microRNA expressions by considering some of them as categorical variables. In particular, we considered: three levels of HbA1c (1 = normal if less than 5.7%, 2 = pre-diabetes if between 5.7 and 6.4%, 3 = diabetes if equal or greater than 6.5%) and two levels of glycemia (1 = normal if less than 126 mg/dL, 2 = high if equal or greater than 126 mg/dL).

Looking at the expression levels of the blue ME across all individuals of our cohort and stratifying them with respect to the specific levels defined for each clinical variable of interest (Fig. 2), we note that it could be feasible to discriminate among HD, OB and OBDM individuals according to the values of these parameters. For example, we found that microRNAs are more expressed in OBDM patients and that these diabetic patients are mainly characterized by higher values of HbA1c, glycemia and HOMA-I (Fig. 2a–d).

#### WGCNA on TP data

**Figure 3:**
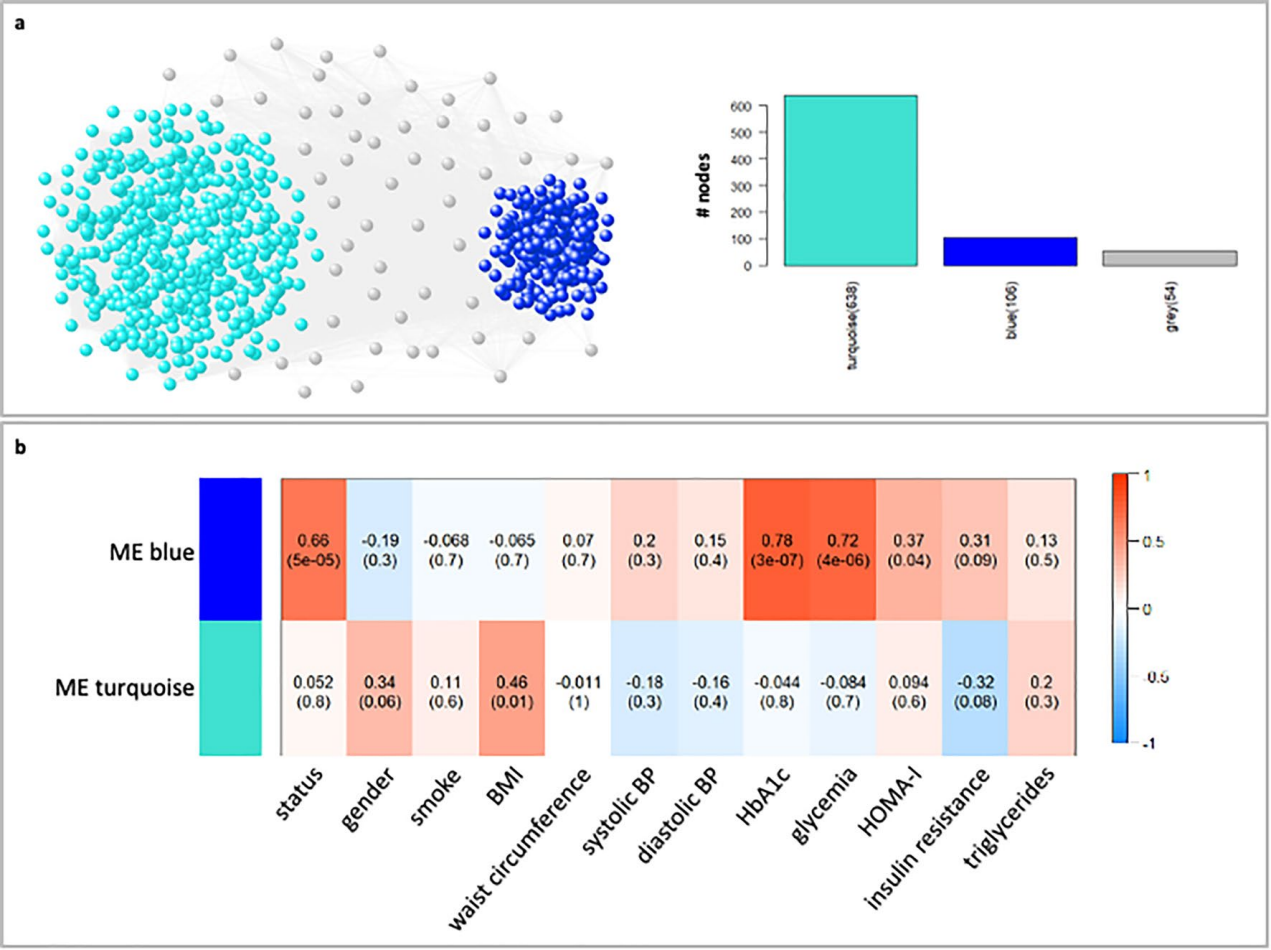
WGCNA analysis on TP data. (**a**) *WGCNA network*. In the correlation network (left), the WGCNA detected modules are highlighted and colored according to the corresponding module labels. Grey color was used to indicate nodes that could not be classified into any module. In the bar plot (right), bars represent the size of each WGCNA detected module and are colored according to the corresponding module labels. (**b**) *Module-trait associations*. In the heatmap, each row corresponds to a module eigengene and each column to a clinical trait of interest. Each cell contains the corresponding correlation and *p*-value. The heatmap is color-coded by correlation according to the color legend.

The WGCNA analysis performed on circulating microRNAs derived from TP led to a co-expression network made of two well-defined modules consisting of 106 and 638 microRNAs (Fig. 3a, Supplementary Table 3).

**Figure 4:**
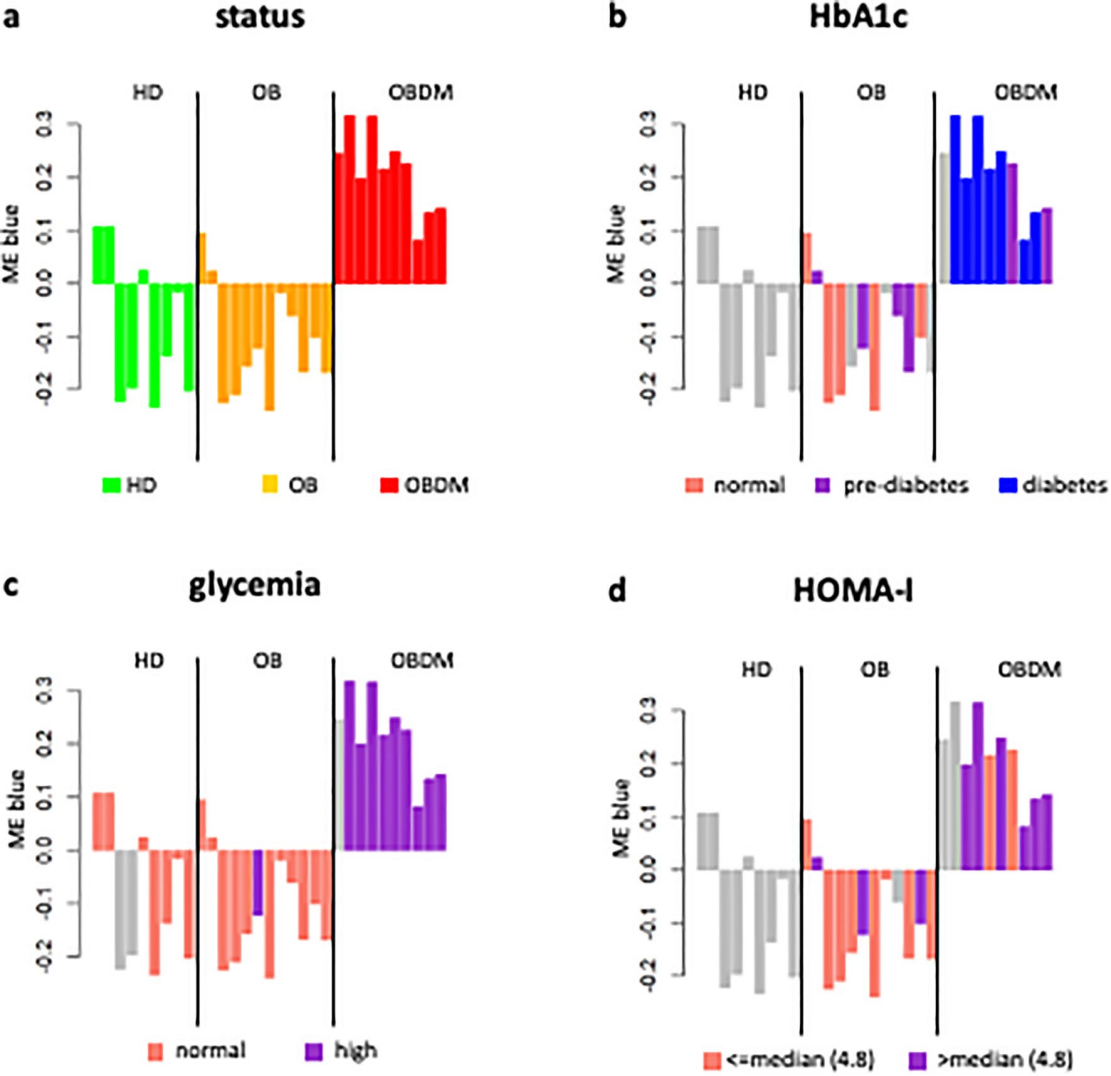
Module-trait association for TP data. Bar plots of the expression levels (y-axis) of blue module eigengene across healthy, OB, and OBDM samples (x-axis). Expression levels of the module eigengenes were log2-transformed and z-score normalized. In each panel, bars were colored according to the stratification used for the specific clinical trait of interest. For HOMA-I, the samples were stratified according to the corresponding median value only for display purpose. Grey was used to indicate not available data.

The heatmap of the module-trait association (Fig. 3b) reinforces the results obtained in the previous analysis on EV data. Indeed, we found again a blue module that exhibits a stronger (positive) and more statistically significant association with the individuals’ status, indicating that microRNAs falling within this module tend to increase from healthy (HD) to diseased condition (OB/OBDM). Yet, the ME of the TP blue module preserves the same correlations and trends of the ME of EV blue module with respect to HbA1c, glycemia and HOMA-I (Figs. 3b and 4a–d).

**Figure 5:**
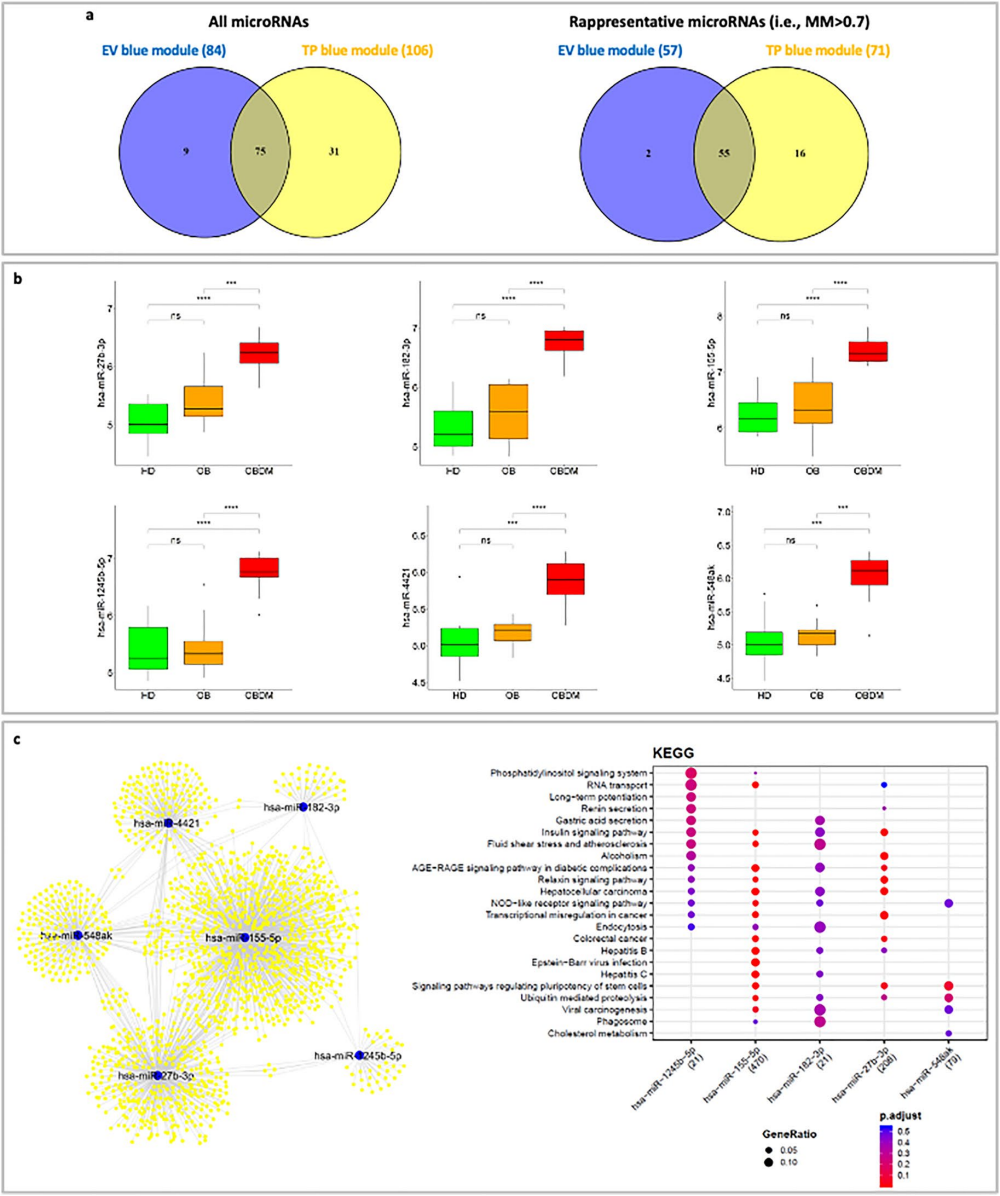
Potential driver microRNAs. (**a**) *Comparison between EV and TP results*. Overlap between the microRNAs in the blue module obtained from EV data and microRNAs in the blue module obtained from TP data. (**b**) *Expression of driver microRNAs in TP*. The boxplots show the gene expression levels (log-transformed) of the six potential driver microRNAs in TP across the HD, OB and OBDM samples. Wilcoxon-test was used to perform pairwise-comparisons and statistical significance was indicated by the star symbols (i.e., ns: *p* > 0.05, *: *p* ≤ 0.05, **: *p* ≤ 0.01, ***: *p* ≤ 0.001, ****: *p* ≤ 0.0001). (**c**) *microRNA-target interaction network and KEGG pathways of driver microRNAs*. The network on the left shows the microRNA-target interactions retrieved from MIENTURNET by querying miRTarBase^52^. These interactions were experimentally validated with strong or weak experimental methods. Blue dots represent microRNAs, yellow dots represent microRNA targets. The panel on the right shows the main KEGG pathways enrichment results for the targets of the microRNAs appearing in the network. These results are presented as a dot plot, where the Y-axis reports the annotation categories (i.e., KEGG pathways) and the X-axis reports the microRNAs with the number of recognized targets (i.e., number of targets with at least one annotation) in round brackets. The colors of the dots represent the adjusted *p*-value s, whereas the size of the dots represents gene ratio (i.e., the number of microRNA targets found annotated in each category over the total number of recognized targets indicated in round brackets). No statistically significant KEGG pathway was detected for miR-4421 targets.

The hypothesis that the TP results may include and even reinforce those of EV is further supported by the evidence that 75 out of 84 microRNAs within the EV blue module are in common with the TP blue module (Fig. 5a). Interestingly, 55 out of the 57 most representative microRNAs of each module are in common, i.e., microRNAs with module membership (MM) greater than 0.7 (Fig. 5a, Supplementary Table 2 and Supplementary Table 3).

Taken together, these findings led us to focus on the blue module of TP and to look for potential driver microRNAs of the patients status within this module. To do this, we firstly ordered microRNAs of TP blue module according to their MM and gene significance (GS). Then, by setting a threshold on the values of these parameters, we selected microRNAs satisfying the conditions MM > 0.7 and a GS > 0.7 (Supplementary Table 2). We ended up with six potential driver microRNAs for further analyses: hsa-miR-27b-3p, hsa-miR-182-3p, hsa-miR-155-5p, hsa-miR-1245b-5p, hsa-miR-4421, hsa-miR-548ak. The boxplots of Fig. 5b show that these microRNAs are overexpressed in OBDM samples and how they are able to discriminate between OBDM and OB or HD, but not between OB and HD (Fig. 5b). This behavior is confirmed also by taking into consideration the EV data (Supplementary Fig. 1). It is worth noting that all driver microRNAs identified from TP are included in the EV blue module, except for hsa-miR-27b-3p, which belongs to the EV turquoise module.

The biological relevance of the six identified driver microRNAs was assessed by creating a network of the experimentally validated microRNA-target interactions and then performing a functional enrichment analysis of the KEGG pathways in which their targets are involved (Fig. 5c and Supplementary Table 4). Interestingly, among the most enriched KEGG pathways, we found “Insulin signaling pathway” and “AGE-RAGE signaling pathway in diabetic complications”, thus suggesting a putative role of the microRNAs of interest in the diabetes development.

### Independent validation patients’ cohort analyses

Validation studies were conducted on an independent group comprising 15 OB and 9 OBDM patients, whose clinical features are reported in Table 2.

**Table 2:** Clinical features of the validation cohort patients’.

	Validation Cohort		
	OB (n = 15)	OBDM (n = 9)	*p*-value
Age	63.36 ± 7.31	69.33 ± 6.03	0.0514
Gender males/females (n)	3/12	2/7	NA
BMI	42.50 ± 8.29	34.97 ± 5.45	0.0243*
Smoking habit Yes/No/Ex (n)	2/10/0	1/6/2	NA
Waist circumference	131.61 ± 8.14	116.67 ± 10.07	0.197
Systolic BP	142.69 ± 23.51	128.22 ± 12.91	0.1798
Diastolic BP	81.15 ± 11.75	78.33 ± 16.33	0.672
Glycemia	93.83 ± 11.57	123.33 ± 32.70	0.0052**
HbA1c	5.83 ± 0.75	6.54 ± 1.26	0.1228
HOMA-I	4.01 ± 2.96	7.60 ± 5.18	0.0796
Insulin resistance Yes/No (n)	7/6	4/1	NA
HDL cholesterol	51.64 ± 15.30	42.00 ± 13.20	0.1355
Triglycerides	137.69 ± 79.59	206.67 ± 134.19	0.145

OB, obese patients; OBDM, obese patients affected by type 2 diabetes (DM2); BMI, body mass index; BP, blood pressure. *p < 0.05, **p < 0.01 OB versus OBDM.

**Figure 6:**
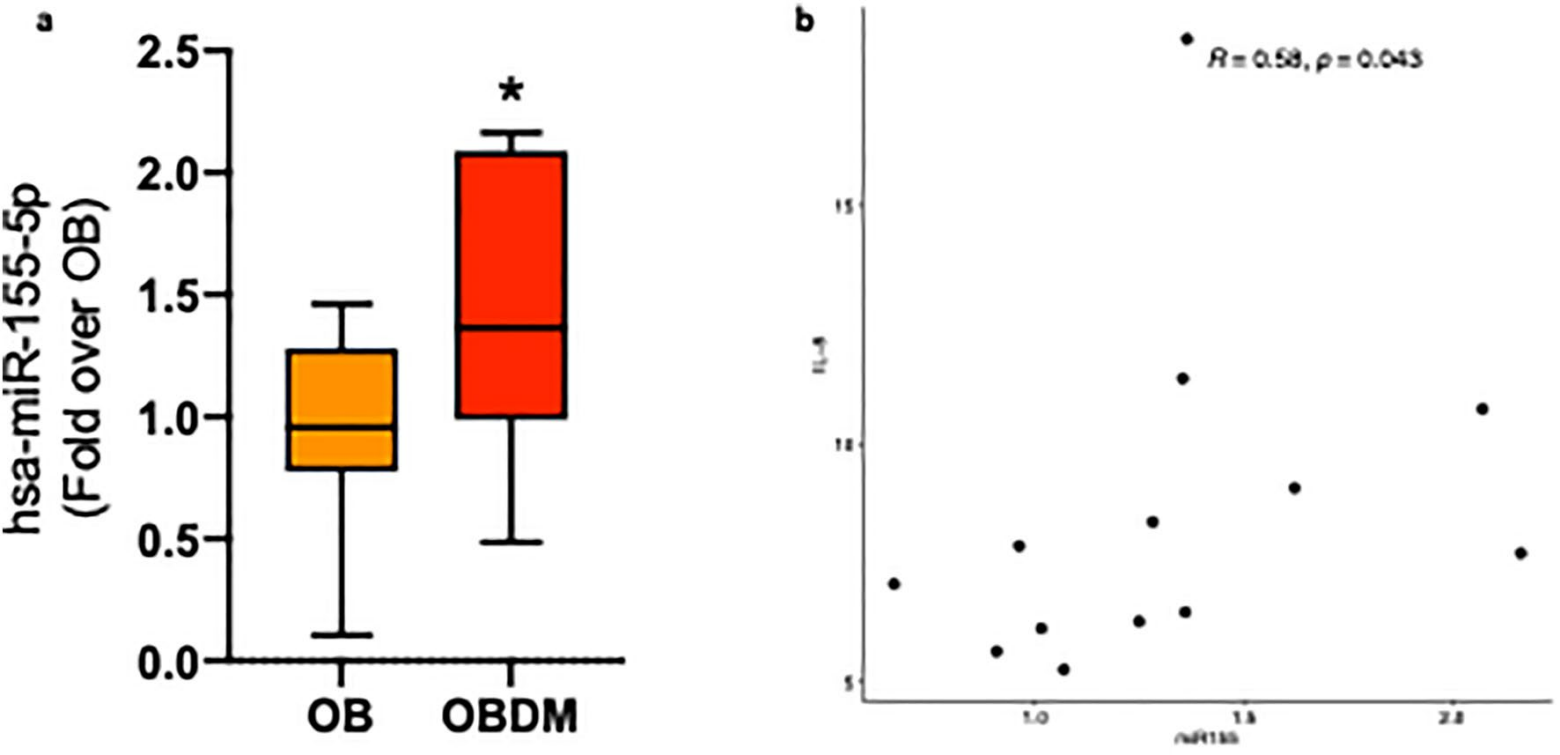
MiR-155-5p confirms its up-regulation in OBDM patients and is correlated with IL-8 levels. (**a**) RT-qPCR analysis was conducted on 15 OB and 9 OBDM patients and miR-155-5p resulted significantly up-regulated in OBDM patients. (**b**) Correlation analysis of miR-155-5p with cytokines resulted in a significant positive correlation with IL-8 levels. *p < 0.05.

For microRNAs validation analysis, the six microRNAs derived from the WGCNA were evaluated by RT-qPCR and miR-155-5p confirmed its up-regulation in OBDM patients (Fig. 6a).

Since miR-155-5p has a key role in inflammation^24^ and obesity and DM2 are considered as chronic inflammatory disorders^25^, 14 cytokines were selected on the basis on their correlation with miR-155-5p, obesity and DM2^25–31^. Interleukin-8 (IL-8) was significantly up-regulated in OBDM patients, conversely leptin was significantly down-regulated (Supplementary Table 5). Intercellular adhesion molecule 1 (ICAM-1), interleukin-1α (IL-1α) and vascular endothelial growth factor (VEGF) were reduced, while the receptor for advanced glycation products (RAGE) was increased in the OBDM group, although not significantly (Supplementary Table 5).

Correlation analyses were conducted to assess possible associations of miR-155-5p expression with both clinical data and cytokines levels. MiR-155-5p showed a significant positive correlation with IL-8 levels (correlation = 0.58, *p*-value = 0.04) (Fig. 6b). Additionally, a multivariate logistic regression model was applied with the aim to find independent predictors of DM2 risk in OB. We found that the combination of miR-155-5p expression levels with IL-8, leptin and RAGE levels was able to significantly predict OB patients at risk of developing DM2 (R^2^ = 0.7, *p*-value = 0.02).

These results underline that miR-155-5p can be used as circulating biomarker for OB patients affected by DM2 and that the combination of miR-155-5p with other inflammatory parameters in OB patients can predict the risk of developing DM2.

## Discussion

In the present study, we profiled both free TP and EVs-embedded circulating microRNAs and used the WGCNA, one of the most employed algorithms that analyzes gene co-expression networks across gene expression data, to explore the relationship between microRNAs and the clinical traits of interest, with the aim of identifying greatly interconnected or co-expressed microRNAs within the weighted network. This approach allowed us to identify six microRNAs able to distinguish between HD and OB individuals with or without DM2. The evaluation of these microRNAs in an extended cohort of OB patients with and without DM2 allowed to confirm miR-155-5p as a circulating biomarker that characterizes OB subjects affected by DM2. Additionally, we identified clinical features and inflammatory markers correlated with miR-155-5p levels and described a model through which the combination of miR-155-5p with IL-8, Leptin and RAGE may be useful to individuate OB patients at higher risk of developing DM2.

MiR-155-5p is one of the most characterized microRNAs. It is hosted in the B-cell integration cluster gene (BIC) in a 13 kb region of the chromosome 21 and is highly expressed by hematopoietic cells, where it can control both erythropoiesis and myelopoiesis, beyond playing pivotal roles in inflammation and immunity^32,33^. MiR-155-5p targets indeed are involved in inflammatory pathways regulation^34^ such as in the control of lipolysis^35,36^, making miR-155-5p an interesting target in chronic inflammatory disorders. Several studies indeed addressed the association between circulating miR-155 dysregulation and DM2. High levels of miR-155 in patients with diabetic nephropathy correlated with microalbuminuria and the combination of serum miR-155 levels with urine vitamin D binding protein had a predictive value in the diagnosis of both onset and poor prognosis of patients with diabetic nephropathy^37^. In contrast, a study conducted on the Iranian population did not report any difference between plasma miR-155 expression in DM2 patients with and without nephropathy and lower levels of plasma miR-155 were detected in DM2 patients compared with healthy subjects^24^. However, the differences among the data of Akhabri, the results of Bai et al. and our results may be due to the different source of samples as well as the exclusion of obese patients from the cohort of the analyzed individuals. In another study conducted on serum of DM2 and healthy Chinese subjects, miR-155 was down-regulated in DM2 patients^38^; the contrasting result may be due again to the different ethnicity of patients included in the study. Additionally, authors did not specify if DM2 patients were also obese. Interestingly, they also used an experimental animal model of transgenic mouse overexpression of miR-155 and determined an improvement in glucose tolerance and insulin sensitivity, underlying the importance of this specific microRNA in glucose metabolism and insulin resistance^38^. Furthermore, Guay et al. demonstrated that miR-155, together with miR-142-3p and miR-142-5p, is transferred by T-lymphocytes derived-exosomes to ß-cells inducing apoptosis and favoring type 1 diabetes development^39^. MiR-155 indeed plays also an important role in both acute and chronic inflammation. It can be modulated by several inflammatory stimuli, such as tumor necrosis factor alpha, interferons as well as pathogen- and damage-associated molecular patterns^40^ and regulates the expression of many inflammatory mediators^41^. We demonstrated that circulating levels of miR-155 are up-regulated in plasma derived from OBDM in respect to OB patients and are positively correlated with IL-8 which, of note, is more abundant in sera of OBDM than in OB patients. Few reports describe the relationship between miR-155 and IL-8. In 2011, Bhattacharyya et al. demonstrated that miR-155 may play a central role in controlling inflammation in cystic fibrosis lung epithelial cells by regulating IL-8 levels through the PI3K/AKT signaling pathway activation^42^. More recently, miR-155 was demonstrated as IL-6 and IL-8 regulator in oral lichen planus (OLS) associated-fibroblasts (OLP AFs). The knockdown of miR-155 indeed determined the reduction of IL-6 and IL-8 release^43^. Conversely, the relationship among miR-155-5p and leptin or RAGE has not been investigated yet. Advanced glycation end products (AGEs) are molecules derived from the reaction of glucose with proteins or lipids that bind to RAGE. The activation of RAGE has been hypothesized to act as a major pathogenic factor in diabetic complications and specifically triggers an increase in cytokines, oxidative stressors and proinflammatory factors, which are involved in cardiovascular alterations. Indeed, miR-155 is also involved in the pathophysiology of cardiovascular diseases^44,45^ and endothelial dysfunction^46,47^. Interestingly, Frati et al. demonstrated that the circulating levels of miR-155 were significantly increased in subjects’ sera after the smoking of just one cigarette^48^ and that the exogenous administration of miR-155 was accompanied by a reduction in VEGF protein levels, corroborating the idea of a role of miR-155 the mechanisms involved in vascular alterations^48^. Of note, we also found a negative correlation between plasma miR-155-5p abundance and VEGF levels in the patients we analyzed.

We are aware that our study has some limitations since it is a monocentric study conducted on a small number of patients, that were recruited, at least in part, during COVID-19 pandemic. However, our data may have important clinical implications since they suggest that miR-155-5p, together with IL-8, Leptin and RAGE, may be used as biomarker to identify obese individuals with higher risk of developing DM2 and cardiovascular complications. Thus, although further studies on a larger group of patients are needed to further corroborate these results, our data support a role for miR-155-5p in chronic inflammation and in the prediction of both metabolic and cardiovascular risk.

## Materials and methods

Unless otherwise stated, commercially available products were used according to the manufacturer’s instructions/protocols.

### Patient cohorts

The study comprised two patients’ groups, a discovery and a validation one.

The discovery group consisted of female and male obese patients without DM2 (OB, n = 12, 56.3 ± 4.6) and obese patients with DM2 (OBDM, n = 10, 62.5 ± 9.0) recruited at Department of Experimental Medicine, Policlinico Umberto I, “Sapienza” University Hospital of Rome. In addition, a cohort of healthy, age- and body mass index (BMI)-matched donors (HD, n = 9, 56.1 ± 4.6) was used as negative control. The validation cohort comprised OB (n = 15, 63.4 ± 7.3) and OBDM (n = 9, 69.3 ± 6.0) patients. Informed written consent was obtained from the patients before enrolment, according to our ethical committee guidelines. Ethical approval (Ref. 5705) was obtained by the Hospital Ethics Committee of “Sapienza” University of Rome, in accordance with the Helsinki declaration of 1964 and its later amendments.

To be eligible for the study, patients should have a diagnosis of obesity (BMI > 30 kg/m^2^ or adipose tissue > 35% in case of women or > 25% in case of men) and/or DM2 (HbA1c ≥ 6.5%). Additionally, women were after menopause. Exclusion criteria were previous bariatric surgery, neoplastic and/or endocrine diseases, liver and/or kidney failure or pharmacological treatments with therapies that could modify the cardio-metabolic and skeletal muscle structures. At the time of enrolment, medical history and physical examination were obtained. Anthropometric/vital parameters, such as weight, height, BMI, blood pressure and biochemical parameters were obtained. Patients’ clinical features are reported in Tables 1 and 2.

### Blood samples processing

Blood samples collected for microRNAs expression were processed within 2 h after collection in BD Vacutainer K2-EDTA tubes and plasma was obtained after centrifugation at 1300 g for 10′ at room temperature (RT). Supernatant was then centrifuged at 1200 g for 20′ at RT and finally at 10,000 g for 30′ at RT. Blood samples for cytokines evaluation were collected in red top vacutainers and processed at the end of clotting time. Serum was obtained after two sequential centrifugations, the first at 1300 g for 10′ at RT and the second at 1200 g for 20′ at RT. Plasma and serum samples were stored at − 80 °C until further use.

### Extracellular vesicles (EVs) isolation

500 µl of plasma were processed with the ExoQuick Plasma prep and Exosome precipitation kit (Cat #EXOQ5TM-1, System Biosciences) to obtain EVs. Briefly, after thrombin addition (final concentration 5U/mL), plasma samples were subsequently centrifuged at 10,000 g for 5′ at RT. Then, supernatants were incubated for 30′ at 4 °C with the Exoquick Exosome Precipitation Solution and then centrifuged at 1500 g for 30′. The resulting pellet was centrifuged again at 1500 g for 5′ and re-suspended with 200 μl of RNase free H_2_O.

### RNA extraction

The automated Maxwell RSC-Promega extractor was used for RNA extraction from TP and EVs by using the Maxwell RSC miRNA Plasma and Serum kit (CAT # AS1680, Promega), following manufacturer’s instructions. The technical quality of the extraction was followed by adding the Ath-miR-159a spike-in during RNA extraction.

### MicroRNA profiling

TP and EVs samples derived from the discovery cohort patients were analysed with the multiplexed NanoString nCounter Human v3 miRNA expression assay (NanoString Technologies, Seattle, WA, USA), as previously described^21^. Briefly, 3 μL RNA derived from TP and EVs was annealed with multiplexed DNA tags (miR-tag) and a ligase enzyme was used to bind mature microRNAs to specific miR-tags, excess was removed by an enzymatic clean-up. After dilution and denaturation, the Reporter and Capture CodeSet were added to 5 μL of the obtained product that was then incubated for 16 h at 70 °C to achieve the hybridization of the Target-Probe Complex. Data collection was performed by using the nCounter Digital Analyzer, where digital images are processed, and the barcode counts are tabulated in a comma separated value format. Raw data quality check and normalization were performed with nSolver 4.0 Software (Nanostring, Seattle, WA, USA).

### RT-qPCR

Spike-in was analyzed with RT-qPCR by using the TaqMan Individual microRNA assays for Ath-miR-159a (code: 000338) (Applied Biosystems, Waltham, MA, USA), as previously described^8^. MicroRNA validation analysis was performed on TP, as already described^49^. MicroRNAs used in the pools are reported in Supplementary Table 1.

### Gene co-expression network analysis

WGCNA is one of the most commonly employed algorithm to construct gene co-expression networks across gene expression data, exploring the association between gene networks and external phenotypic/clinical traits of interest^22,23^.

Briefly, WGCNA first builds a weighted network where nodes correspond to genes and edges are weighted according to the pairwise correlations between their gene expressions. Then, WGCNA identifies modules of highly interconnected, or co-expressed, genes within the weighted network by grouping together the most similar nodes. The similarity measure between two nodes is expressed in terms of their direct connection strength as well as connection strengths “mediated” by shared neighbors. The relationship between modules as well as with the external traits can be studied by exploiting the so-called module eigengene (ME). The ME is defined as the first principal component of a given module and can be considered a representative of the gene expression profiles in that module. The relevance of each gene is assessed by computing two parameters: the module membership (MM) and the gene significance (GS). The MM is defined as the correlation between the gene expression profile and the ME of a given module. If MM of a given gene with respect to a given module is close to 0, that gene is not part of that module. On the other hand, if MM is close to 1 or -1, the gene is highly connected to the genes of that module. The sign of MM encodes whether the gene has a positive or a negative relationship with the ME. The GS is defined as the correlation between the gene expression profile and a given external sample trait. Abstractly speaking, the higher the absolute value of GS of a given gene, the more biologically significant is that gene. The gene significance of 0 indicates that the gene is not significant with regard to the biological question of interest. The GS can take on positive or negative values. In the present study, potential driver microRNAs were identified as those microRNAs of a given module highly connected within the module (highest MM in absolute value) and most strongly correlated with the trait of interest (highest GS in absolute value). Both for MM and GS, we selected a threshold equal to 0.7.

### MicroRNA-target interaction network

The microRNA-target interaction networks were constructed by exploiting MIENTURNET (MicroRNA ENrichment TURned NETwork)^50^, a web tool designed to receive in input a list of microRNAs and infer possible evidences of their regulation on target genes, based on both statistical and network-based analyses. In particular, MIENTURNET produces a network where nodes are microRNAs and target genes and a link occurs between them if an interaction among them is computationally predicted and/or experimentally validated from TargetScan and miRTarBase, respectively.

### Functional enrichment analysis

The functional enrichment analysis was performed by querying Kyoto Encyclopedia of Genes and Genomes (KEGG)^51^ pathway through MIENTURNET web tool^50^. *p*-value s were adjusted with the Benjamini–Hochberg method and a threshold equal to 0.05 was set to identify functional annotations significantly enriched amongst genes of the input list.

### Cytokines evaluation

Fourteen cytokines were selected (HGF, ICAM1, IFN-γ, IL-10, IL-17, IL-1-α, IL-1- β, IL-6, IL-8, Leptin, Rage, Resistin, TNF-α, VEGF) and measured on serum samples belonging to the validation cohort patients by BioPlex (Luminex Technology, BioRad).

### Statistical analysis

Statistical analyses were performed using GraphPad Prism Software version 9.0 (La Jolla, California, USA). Student’s unpaired t-test was used to determine significant differences between microRNAs or cytokines in the validation cohort. Correlation analyses were performed by using non-parametric Spearman's rank test through R statistical software (version 4.1.0). A multivariate logistic regression model was constructed to evaluate the relationships between the outcome of interest (OB/OBDM status) and the predicted biomarkers (microRNAs and cytokines). In all statistical analyses, *p*-value < 0.05 was considered statistically significant.

### Supplementary Information

Supplementary Figure 1.

Supplementary Table 1.

Supplementary Table 2.

Supplementary Table 3.

Supplementary Table 4.

Supplementary Table 5.

## Data Availability

The authors confirm that the data supporting the findings of this study are available within the article [and/or] its supplementary materials.

## References

[1] Lancet T (2017). Diabetes: a dynamic disease. Lancet.

[2] Pescador N (2013). Serum circulating microRNA profiling for identification of potential type 2 diabetes and obesity biomarkers. PLoS ONE.

[3] Kim H (2020). Effect of diabetes on exosomal miRNA profile in patients with obesity. BMJ Open Diabetes Res. Care.

[4] Kwon SH (2020). Changes in kidney function markers after bariatric surgery in morbidly obese patients. Kidney Res. Clin. Pract..

[5] Polyzos SA, Kountouras J, Mantzoros CS (2019). Obesity and nonalcoholic fatty liver disease: From pathophysiology to therapeutics. Metabolism.

[6] Zimmet PZ (2017). Diabetes and its drivers: The largest epidemic in human history?. Clin. Diabetes Endocrinol..

[7] Ha M, Kim VN (2014). Regulation of microRNA biogenesis. Nat. Rev. Mol. Cell Biol..

[8] Catanzaro G (2018). Circulating microRNAs in elderly type 2 diabetic patients. Int. J. Endocrinol..

[9] Catanzaro G (2021). Tissue and circulating microRNAs as biomarkers of response to obesity treatment strategies. J. Endocrinol. Investig..

[10] Ciuffi S (2022). Circulating microRNAs as biomarkers of osteoporosis and fragility fractures. J. Clin. Endocrinol. Metab..

[11] Filardi T (2020). Non-coding RNA: Role in gestational diabetes pathophysiology and complications. Int. J. Mol. Sci..

[12] Brandão-Lima PN (2022). Circulating microRNAs showed specific responses according to metabolic syndrome components and sex of adults from a population-based study. Metabolites.

[13] Agarwal V, Bell GW, Nam J-W, Bartel DP (2015). Predicting effective microRNA target sites in mammalian mRNAs. elife.

[14] Ye D (2018). Plasma miR-17, miR-20a, miR-20b and miR-122 as potential biomarkers for diagnosis of NAFLD in type 2 diabetes mellitus patients. Life Sci..

[15] Veitch S (2022). MiR-30 promotes fatty acid beta-oxidation and endothelial cell dysfunction and is a circulating biomarker of coronary microvascular dysfunction in pre-clinical models of diabetes. Cardiovasc. Diabetol..

[16] Solís-Toro D, Escudero MM, García-Perdomo HA (2022). Association between circulating microRNAs and the metabolic syndrome in adult populations: A systematic review. Diabetes Metab. Syndr. Clin. Res. Rev..

[17] Villard A, Marchand L, Thivolet C, Rome S (2015). Diagnostic value of cell-free circulating microRNAs for obesity and type 2 diabetes: a meta-analysis. J. Mol. Biomark. Diagn..

[18] de Gonzalo-Calvo D (2017). Serum microRNA-1 and microRNA-133a levels reflect myocardial steatosis in uncomplicated type 2 diabetes. Sci. Rep..

[19] Zampetaki A (2012). Prospective study on circulating MicroRNAs and risk of myocardial infarction. J. Am. College Cardiol..

[20] Mahjoob G, Ahmadi Y, Fatima Rajani H, Khanbabaei N, Abolhasani S (2022). Circulating microRNAs as predictive biomarkers of coronary artery diseases in type 2 diabetes patients. J. Clin. Lab. Anal..

[21] Bye A (2016). Circulating microRNAs predict future fatal myocardial infarction in healthy individuals—The HUNT study. J. Mol. Cell. Cardiol..

[22] Langfelder P, Horvath S (2008). WGCNA: An R package for weighted correlation network analysis. BMC Bioinform..

[23] Zhang B, Horvath S (2005). A general framework for weighted gene co-expression network analysis. Stat. Appl. Genet. Mol. Biol..

[24] Akhbari M, Khalili M, Shahrabi-Farahani M, Biglari A, Bandarian F (2019). Expression level of circulating cell free miR-155 gene in serum of patients with diabetic nephropathy. Clin. Lab..

[25] Baldeón RL (2014). Decreased serum level of miR-146a as sign of chronic inflammation in type 2 diabetic patients. PloS One.

[26] Choi S (2017). Carbon monoxide prevents TNF-α-induced eNOS downregulation by inhibiting NF-κB-responsive miR-155-5p biogenesis. Exp. Mol. Med..

[27] Elisia I (2020). The effect of smoking on chronic inflammation, immune function and blood cell composition. Sci. Rep..

[28] Kay AM, Simpson CL, Stewart JA (2016). The role of AGE/RAGE signaling in diabetes-mediated vascular calcification. J. Diabetes Res..

[29] Ksiazek-Winiarek D, Szpakowski P, Turniak M, Szemraj J, Glabinski A (2017). IL-17 exerts anti-apoptotic effect via miR-155-5p downregulation in experimental autoimmune encephalomyelitis. J. Mol. Neurosci..

[30] Piperi C, Goumenos A, Adamopoulos C, Papavassiliou AG (2015). AGE/RAGE signalling regulation by miRNAs: associations with diabetic complications and therapeutic potential. Int. J. Biochem. Cell Biol..

[31] Tang X (2020). The miR-155 regulates cytokines expression by SOSC1 signal pathways of fish in vitro and in vivo. Fish Shellfish Immunol..

[32] Elton TS, Selemon H, Elton SM, Parinandi NL (2013). Regulation of the MIR155 host gene in physiological and pathological processes. Gene.

[33] Jankauskas SS, Gambardella J, Sardu C, Lombardi A, Santulli G (2021). Functional role of miR-155 in the pathogenesis of diabetes mellitus and its complications. Non-Coding RNA.

[34] Cai X (2012). Re-polarization of tumor-associated macrophages to pro-inflammatory M1 macrophages by microRNA-155. J. Mol. Cell Boil..

[35] Liu S, Yang Y, Wu J (2011). TNFα-induced up-regulation of miR-155 inhibits adipogenesis by down-regulating early adipogenic transcription factors. Biochem. Biophys. Res. Commun..

[36] Hou L, Chen J, Zheng Y, Wu C (2016). Critical role of miR-155/FoxO1/ROS axis in the regulation of non-small cell lung carcinomas. Tumor Biol..

[37] Bai X, Luo Q, Tan K, Guo L (2020). Diagnostic value of VDBP and miR-155-5p in diabetic nephropathy and the correlation with urinary microalbumin. Exp. Therap. Med..

[38] Lin X (2016). MiR-155 enhances insulin sensitivity by coordinated regulation of multiple genes in mice. PLoS Genet..

[39] Guay C (2019). Lymphocyte-derived exosomal microRNAs promote pancreatic β cell death and may contribute to type 1 diabetes development. Cell Metab..

[40] Mahesh G, Biswas R (2019). MicroRNA-155: A master regulator of inflammation. J. Interferon Cytokine Res..

[41] Marques-Rocha JL (2015). Noncoding RNAs, cytokines, and inflammation-related diseases. FASEB J..

[42] Bhattacharyya S (2011). Elevated miR-155 promotes inflammation in cystic fibrosis by driving hyperexpression of interleukin-8. J. Biol. Chem..

[43] Cheng J (2022). MiR-155-5p modulates inflammatory phenotype of activated oral lichen-planus-associated-fibroblasts by targeting SOCS1. Mol. Biol. Rep..

[44] Cao RY (2016). The emerging role of microRNA-155 in cardiovascular diseases. BioMed Res. Int..

[45] Welten S, Goossens E, Quax P, Nossent A (2016). The multifactorial nature of microRNAs in vascular remodelling. Cardiovasc. Res..

[46] Sun H-X (2012). Essential role of microRNA-155 in regulating endothelium-dependent vasorelaxation by targeting endothelial nitric oxide synthase. Hypertension.

[47] Liu Y (2015). MicroRNA-155 regulates ROS production, NO generation, apoptosis and multiple functions of human brain microvessel endothelial cells under physiological and pathological conditions. J. Cell. Biochem..

[48] Frati G (2020). inhibition of miR-155 attenuates detrimental vascular effects of tobacco cigarette smoking. J. Am. Heart Assoc..

[49] Filardi T (2022). Identification and validation of miR-222-3p and miR-409-3p as plasma biomarkers in gestational diabetes mellitus sharing validated target genes involved in metabolic homeostasis. Int. J. Mol. Sci..

[50] Licursi V, Conte F, Fiscon G, Paci P (2019). MIENTURNET: An interactive web tool for microRNA-target enrichment and network-based analysis. BMC Bioinform..

[51] Kanehisa M, Sato Y, Kawashima M, Furumichi M, Tanabe M (2016). KEGG as a reference resource for gene and protein annotation. Nucleic Acids Res..

[52] Chou C-H (2016). miRTarBase 2016: Updates to the experimentally validated miRNA-target interactions database. Nucleic Acids Res..

