# Supplementary material for: Network analysis identifies circulating miR-155 as predictive biomarker of type 2 diabetes mellitus development in obese patients: a pilot study
Source: Sci Rep. 2023 Nov 9;13:19496. doi: 10.1038/s41598-023-46516-y (PMC10636008; doi:10.1038/s41598-023-46516-y)

Supplementary Figure 1. Expression of driver microRNAs in EV. The boxplots show the gene expression levels (log-transformed) of the six potential drivers microRNAs in EV across the HD, OB and OBDM samples. Wilcoxon-test was used to perform pairwise-comparisons and statistical significance was indicated by the star symbols (i.e., ns: $p > 0.05$, *: $p \le 0.05$, **: $p \le 0.01$, ***: $p \le 0.001$, ****: $p \le 0.0001$).

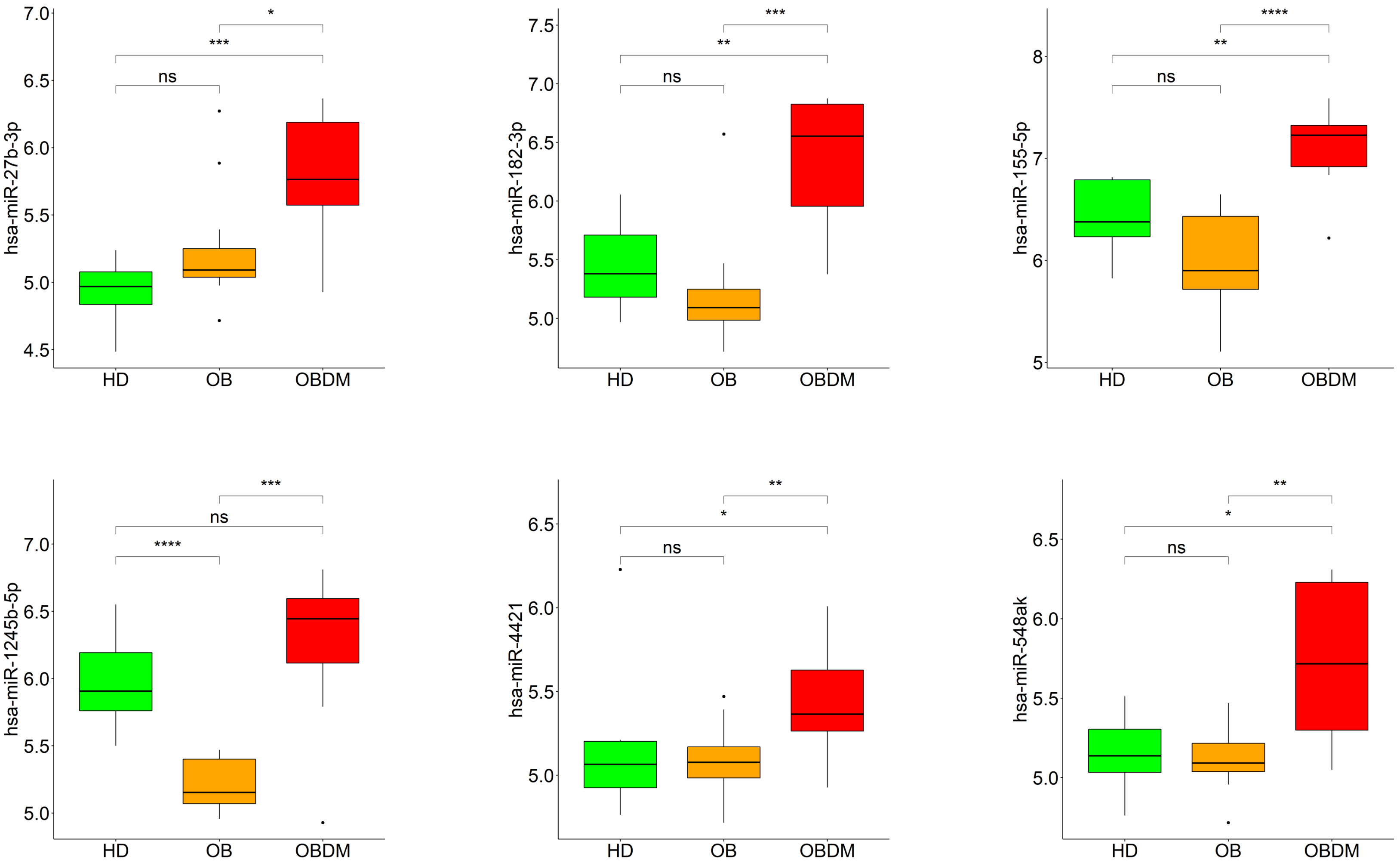

Supplement: Supplementary file 1 — Supplementary Figure 1. [file 41598_2023_46516_MOESM1_ESM.pdf]
