# Supplementary material for: Network analysis identifies circulating miR-155 as predictive biomarker of type 2 diabetes mellitus development in obese patients: a pilot study
Source: Sci Rep. 2023 Nov 9;13:19496. doi: 10.1038/s41598-023-46516-y (PMC10636008; doi:10.1038/s41598-023-46516-y)
Supplement: Supplementary file 6 — Supplementary Table 5. [file 41598_2023_46516_MOESM6_ESM.docx]

**Supplementary Table 5.** Serum cytokines levels in OB and OBDM patients.

|  | **OB** | **OBDM** | **p-value** |
| --- | --- | --- | --- |
| **HGF** | 285.53 ± 90.02 | 279.88 ± 86.15 | 0.8717 |
| **ICAM1** | 801100.97 ± 493343.90 | 584783.31 ± 409001.40 | 0.1166 |
| **IL-1A** | 49.43 ± 27.75 | 39.44 ± 21.96 | 0.3157 |
| **IL-8** | 6.51 ± 1.06 | 10.20 ± 3.95 | 0.0023** |
| **LEPTIN** | 96960.22 ± 29970.47 | 59861.60 ± 42201.83 | 0.0089** |
| **RAGE** | 2289.65 ± 585.18 | 2721.79 ± 1649.30 | 0.1991 |
| **RESISTIN** | 15558.86 ± 4119.66 | 15765.16 ± 10149.48 | 0.4741 |
| **TNF-A** | 8.55 ± 1.18 | 9.86 ± 4.86 | 0.1865 |
| **VEGF** | 113.77 ± 81.56 | 73.13 ± 49.85 | 0.0236* |

OB, obese patients; OBDM, obese patients affected by type 2 diabetes (DM2).

* p<0.05, **p<0.01 OB *vs* OBDM.
